# The Coordination and Activity Tracking in CHildren (CATCH) study: rationale and design

**DOI:** 10.1186/s12889-015-2582-8

**Published:** 2015-12-21

**Authors:** John Cairney, Cheryl Missiuna, Brian W. Timmons, Christine Rodriguez, Scott Veldhuizen, Sara King-Dowling, Sarah Wellman, Tuyen Le

**Affiliations:** Department of Family Medicine, McMaster University, David Braley Health Sciences Centre, 100 Main Street West, 5th Floor, Hamilton, ON L8P 1H6 Canada; School of Rehabilitation Science and CanChild, McMaster University, 1280 Main Street West, Hamilton, ON L8S 4K1 Canada; Department of Pediatrics, McMaster University, 1280 Main Street West, Hamilton, ON L8S 4K1 Canada; Department of Kinesiology, McMaster University, 1280 Main Street West, Hamilton, ON L8S 4K1 Canada; INfant and Child Health (INCH) Lab, Department of Family Medicine, McMaster University, David Braley Health Sciences Centre, 100 Main Street West, 1st Floor, Hamilton, L8P 1H6 Canada

**Keywords:** Body composition, Developmental coordination disorder, Early years, Health-related fitness, Movement difficulties, Motor skills, Obesity

## Abstract

**Background:**

Past studies have found that children with Developmental Coordination Disorder (DCD) engage in less physical activity than typically developing children. This “activity deficit” may result in children with DCD being less physically fit and more likely to be overweight or obese, potentially increasing later risk for poor cardiovascular health. Unfortunately, the majority of DCD research has been limited to cross-sectional designs, leading to questions about the complex relationship among motor ability, inactivity and health-related fitness. Of the few longitudinal studies on the topic, determining precedence amongst these factors is difficult because study cohorts typically focus on mid to late childhood. By this age, both decreased physical fitness and obesity are often established. The Coordination and Activity Tracking in CHildren (CATCH) study will examine the pathways connecting DCD, physical activity, physical fitness, and body composition from early to middle childhood.

**Methods:**

The CATCH study is a prospective cohort study. We aim to recruit a cohort of 600 children aged 4 to 5 years (300 probable DCD [pDCD] and 300 controls) and test them once a year for 4 years. At Phase 1 of baseline testing, we assess motor skills, cognitive ability (IQ), basic anthropometry, flexibility and lower body muscle strength, while parents complete an interview and questionnaires regarding family demographics, their child’s physical activity, and behavioural characteristics. Children who move on to Phase 2 (longitudinal cohort) have their body fat percentage, foot structure, aerobic and anaerobic fitness assessed. An accelerometer to measure physical activity is then given to the child and interested family members. The family also receives an accelerometer logbook and 3-day food dairy. At years 2 to 4, children in the longitudinal cohort will have all baseline assessments repeated (excluding the IQ test), and complete an additional measure of perceived self-efficacy. Parents will complete an ADHD index twice within the follow-up period. To assess the association between DCD, fitness and adiposity, our primary analysis will involve longitudinal growth models with fixed effects.

**Discussion:**

The CATCH study will provide a clearer understanding of pathways between DCD and health-related fitness necessary to determine the types of interventions children with DCD require.

## Background

Developmental coordination disorder (DCD) is a prevalent and serious neuro-developmental condition characterized by problems with fine and/or gross motor coordination that result in impairment in everyday functioning, play, and academic achievement [1, 2]. On average, DCD affects 1 to 2 children in each classroom [3]. Although there are clear diagnostic criteria [1, 4], DCD is seldom recognized or diagnosed [5]. As a result, the difficulties experienced by children at school and at home are often mistakenly ascribed to oppositional behaviour, learning or attention difficulties, or simple laziness [5, 6].

In addition to academic and self-care difficulties, children with DCD have repeatedly been shown to be less physically active than other children [7, 8]. One hypothesis in the field is that because of this “activity deficit” [9], children with DCD become less physically fit than typically developing children and are more likely to be overweight or obese [10, 11]. If true, this poses a serious problem for children with DCD, because they would be at a greatly increased risk for poor cardiovascular health and, in the long term, for early onset of chronic disease [12]. In addition, recent evidence of a link between inactivity and delayed skeletal development has now also raised concern about other potential health consequences, such as increased risk of fractures and injuries [13]. Testing the idea of an activity deficit also has the potential to clarify what types of interventions might be most appropriate for these children.

To date, most research on physical health and DCD has used cross-sectional study designs and has therefore not examined the complex associations among DCD, inactivity and health-related fitness over time [14–18]. At best, cross-sectional studies can only suggest causal associations among DCD, physical activity, physical fitness, and obesity. Among the few studies that have used longitudinal designs [19–21], determining the precedence of each of these factors has been complicated by the fact that data collection started in middle childhood (ages 8 years and older). By this age, both decreased physical fitness and obesity are often established [20–22]. Another limitation of previous longitudinal research is that motor coordination has been assessed at only one time using a single motor test *and* without considering other diagnostic criteria [19–21]. While the motor impairments that are at the core of DCD are believed to be stable and chronic, this is a largely untested assumption. Poor motor skill development may lead to withdrawal from physical activity and subsequently, to decreased fitness and overweight/obesity, conditions that are potentially amenable to change. However, it is equally plausible that low strength and endurance, and even obesity, could be features of the developmental syndrome that inevitably emerge over time.

The goal of the Coordination and Activity Tracking in CHildren (CATCH) study is to examine the pathways connecting DCD, physical activity, physical fitness, and body composition from early to middle childhood. These relationships are important in order to determine the types of interventions children with DCD require. This prospective cohort study will follow 4- and 5-year old boys and girls for 4 years with annual assessments of physical activity, body composition, health-related fitness, and motor skills.

Specifically, our objectives are:

1. To examine the stability of DCD over time. We hypothesize that children who meet study criteria for DCD at baseline will continue to meet criteria for DCD at follow-up assessments, and that children who do not meet study criteria for DCD at baseline will not meet criteria in later assessments.
2. To evaluate the precedence of DCD, fitness and adiposity. We hypothesize that children who meet criteria for DCD at baseline will, over time, show decreasing aerobic and anaerobic fitness and increasing weight (Body Mass Index [BMI], body fat percentage and waist circumference) relative to children who are typically developing. This divergence will be partially mediated by differences in physical activity.

## Methods/Design

### Study design

The CATCH study is a prospective cohort study with an initial cohort of 600 children aged 4 or 5 years at baseline: 300 with probable DCD (pDCD) and 300 controls. Using the Optimal Design software package [23], we have determined that an analysis with two groups of 300 assessed on 4 occasions will permit the detection of standardized effect size differences of approximately 0.3 with 80 % power and a significance level of 0.05. Taking expected attrition into account (~10 % per year), standardized effect sizes above approximately 0.35 will remain detectable. This means with 600 participants, we will be adequately powered to detect modest effect size differences between our two groups while accounting for loss to follow-up over time. The control group is randomly selected from the children completing the laboratory assessment who do not meet criteria for DCD. Data collection occurs at annual study visits, which include a motor coordination assessment, anthropometric assessments, and non-invasive physical fitness measures. All measures have previously been conducted successfully in this age group [24–26].

Through a parental interview, DCD questionnaire and demographic survey, parents provide additional information about their child’s performance of daily motor activities, demographics, measures of health and risk factors for obesity (adapted from surveys exploring these risk factors) and measures of participation in physical activity [27, 28]. The Hamilton Integrated Research Ethics Board has provided ethical approval for the study*.*

### Participants

The target age range for participants is 48 months, 0 days to 71 months, 30 days at study entry. Children with a physical disability or diagnosed medical condition that affects motor coordination (e.g. Cerebral Palsy) are excluded from the study. Children with a birth weight lower than 1500 g are also not eligible. These eligibility criteria are necessary in order to rule out medical conditions other than DCD, which may be responsible for poor motor coordination.

#### Recruitment strategy

The sample is drawn from various community organizations and sites within the city of Hamilton, Ontario and surrounding area. These include parenting resource and support centres (e.g. Ontario Early Years Centres, Best Start programs), kindergarten classes in the public and Catholic school boards, among others.

Parents who provide their contact information via partnering community sites or contact the study team directly are telephoned by the research staff to determine their child’s eligibility and obtain informed, verbal consent. Eligible parents and children who provide verbal consent are invited to participate in the Phase 1 baseline laboratory visit. The recruitment process involves one randomization to select a proportion of Movement Assessment Battery for Children (M-ABC-2)-negative (typically developing) children into the control arm in the longitudinal study.

**Fig. 1:**
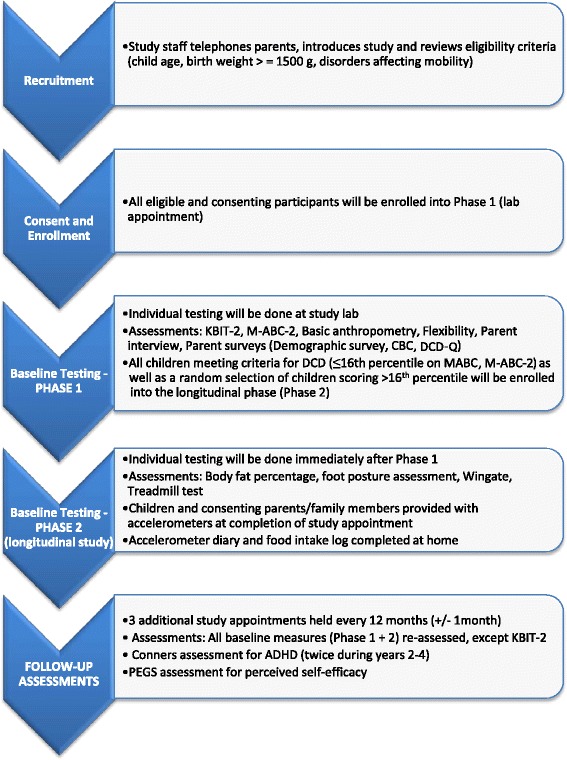
Recruitment and study protocol. ADHD, Attention Deficit Hyperactivity Disorder; DCD-Q, Developmental Coordination Disorder Questionnaire; KBIT-2, The Kaufman Brief Intelligence Test 2^nd^ Edition; M-ABC-2, Movement Assessment Battery for Children 2^nd^ Edition; CBCL, Child Behaviour Checklist; PEGS, Perceived Efficacy and Goal Setting System

After Phase 1 of baseline testing, all children positive on the M-ABC-2 (pDCD) are recruited to the longitudinal study. Children who are negative on the M-ABC-2 are randomized to determine if they will continue onto Phase 2 (Fig. 1). Randomization probabilities are calculated to produce a sample of controls with a distribution of motor functioning that matches the population distribution of non-cases.

#### Assembling the study cohort at baseline

As noted previously, we will assemble a cohort of 600 children for the longitudinal portion of the study: 300 children meeting criteria for pDCD (see below) and 300 typically developing children. This approach will provide a balanced sample, which maximizes statistical power at a given sample size. We will use random sampling rather than matching because this approach ensures that this group is representative of typically developing children with respect to both measured and unmeasured variables [29].

#### Defining cases of pDCD

We will define cases based, in part, on the 2011 European Academy of Childhood Disability (EACD) Guidelines for Identification of Children with DCD [30], which require: (1) a score at or below the 16^th^ percentile on a standardized measure of motor impairment (M-ABC-2) [31]; (2) evidence of impact on daily function; (3) IQ score above 70 (KBIT-2) [32]; (4) absence of any medical condition affecting motor functioning (parent reported). In addition, we have also decided to exclude children whose birth weight is less than 1500 grams, as children with very low birth weight are apt to present with a variety of co-morbid health problems that may affect motor coordination. The EACD guidelines also recommend repeated measurement of motor function in children under 6 years of age to ensure valid and reliable identification of children with DCD, which we will also do.

### Measurements

#### Motor coordination

A standardized motor coordination assessment is conducted in order to identify children with pDCD. We are using the Movement Assessment Battery for Children 2^nd^ Edition (M-ABC-2), the most widely used assessment for the identification of DCD [26, 33, 34]. The M-ABC-2 is an individually administered standardized test which includes 8 motor tasks within 3 categories: Manual Dexterity; Aiming & Catching; and Balance (static and dynamic). Raw scores on these items are converted into standard scores based on the child’s age, and then converted into an overall percentile. Test-retest reliability and standard of error of measurement for the total test scores have been reported to be 0.80 and 1.34, respectively [31]. Studies have shown that the M-ABC-2 is a reliable and valid tool for the assessment of movement difficulties, even in very young children (4 to 6 years of age) [31].

#### Performance of daily activities

Questionnaires to assess the impact of DCD on everyday activities have not yet been validated for this age. Therefore, a published parental semi-structured interview (developed by our team) is used to ask parents how their child’s motor coordination difficulties affect active play, self-care, and time spent at pre-school/school. It has been used successfully in studies with children 4 years and older to confirm DCD [35, 36]. The interview is also administered to parents of typically developing children. For a child to be considered to have DCD, parents must report evidence of significant functional impact in at least one domain (e.g. self-care).

#### Intellectual ability

The Kaufman Brief Intelligence Test 2^nd^ Edition (KBIT-2) is administered only at baseline. This is to ensure all children included in the study have IQs > 70. It is a standardized measure of intelligence that has been used in large studies to estimate children’s cognitive ability. The KBIT-2 measures function in two cognitive domains (verbal and non-verbal) and is a reliable measure that requires no reading or writing and is suitable for children 4 years of age and older [37].

#### Body composition analysis

Basic anthropometric assessments are conducted. Standing and sitting height are measured without shoes to the nearest 0.1 cm using a calibrated portable stadiometer (Seca 213) and body mass is measured in light clothing without shoes to the nearest 0.1 kg using a digital scale (Seca 869). From this, BMI can be calculated. In order to assess body fat distribution, specifically central adiposity, waist circumference (WC) is measured to the nearest 0.1 cm at 2 sites (the uppermost lateral border of the iliac crest, and the midpoint between the last floating rib and the top of the iliac crest) [38]. WC has been shown to correlate well (*r* = 0.84) [39–41] with trunk fat mass measured by duel-energy x-ray absorptiometry and to be a sensitive marker of future cardiovascular risk in young children [42]. If the two height and weight measures differ by more than 0.1 cm (0.4 cm for sitting height) or 0.1 kg, additional measures are taken until there are two that differ by ≤0.1 cm (0.4 cm for sitting height) or ≤0.1 kg, respectively. If the two WC measures differ by more than 0.5 cm, additional measures are taken until there are two that differ by ≤0.5 cm. The average of the two closest measures is used for height, weight and WC. Body composition is also assessed using bioelectrical impedance analysis (BIA) (RJL Systems – Quantum IV Body Composition Analyzer) while the child is in a supine position. BIA is a simple, non-invasive method to estimate percent body fat by passing a low level electrical current through the body [43]. Fat-free mass (FFM) will be calculated using an age-specific equation [44] validated against the doubly-labelled water technique. Percent body fat is then calculated as [(Body weight – FFM)/body weight] ×100.

#### Foot structure

Previous work examining the relationships between foot structure, balance and weight in children has found differences between obese and non-obese children. Children who are obese have flatter feet [45, 46] than do children of normal weight. For individuals with flatter feet, associations have been found suggesting they have more health problems and poorer balance [46]. Much of this work, however, has been cross-sectional and has not examined the precedence of each of these factors. Foot structure is measured using the Foot Posture Index Six-item version (FPI-6), which quantifies the degree to which a foot can be considered to be in a normal or abnormal position [47]. This assessment is taken with the child standing in a relaxed, weight-bearing and static stance posture. The FPI-6 measures (1) talar head palpation; (2) supra- and infra-lateral malleolar curvature; (3) calcaneal frontal plane position; (4) prominence in the region of the talonavicular joint; (5) congruence of the medial longitudinal arch; and (6) abduction/adduction of the forefront on rearfoot. A 5-point scale (−2, −1, 0, 1, 2) is used to score each item, where the negative and positive scores correspond to supinated and pronated postures, respectively, with zero representing a neutral position. The finalized 6-item version has been found to predict 64 % of the variance in static standing posture and 41 % of the variance in midstance posture during normal walking [47].

#### Physical fitness

Both aerobic and anaerobic fitness are assessed. Aerobic fitness is assessed on a treadmill (Valiant, LODE) using a progressive exercise test. The Bruce protocol is used; the multistage test begins with the participant walking slowly for 3 min at 2.7 km/h at 10 % grade [44]. Participants complete the full Bruce protocol as it has been shown to provide a more accurate measure of maximal endurance time (time to exhaustion) in 4- and 5-year-olds than a modified Bruce protocol. To assist with coordination and balance, participants are instructed to hold onto the handrails for the duration of the test. As well, a researcher is positioned directly behind the child to ensure safety. Heart rate is assessed continuously using a monitor (Polar Electro FT1, Kempele, Finland) worn by the child around the chest. As the speed and incline of the treadmill increase (every 3 min), the child is encouraged to stay walking or running on the treadmill for as long as possible. The test is terminated when the participant is no longer able to continue with the increasing speed or incline of the treadmill, or with refusal to continue exercising despite verbal encouragement. On completion of the treadmill test, the child remains seated quietly for 2 min with heart rate being recorded continuously (approximately 5 times/s) during this recovery period. Both time to exhaustion and heart rate recovery (HRR) are used as indices of aerobic fitness [48]. 1 min HRR is calculated as the difference between HR at the end of the test and at 1 min during recovery. Measurements of HRR using a similar protocol have shown to be feasible and reliable in our pilot work [24, 25].

To estimate short-term muscle power (anaerobic fitness), children complete a Wingate anaerobic cycling test using a cycle ergometer (Pediatric Corival, LODE). To determine maximum pedaling speed, the child sprints as fast as they can for approximately 30 s, pedalling only against the internal resistance of the ergometer. After a short rest, the child begins the Wingate test. When the child reaches 80 % of their maximum pedalling speed, the braking force is applied (0.55 N.m.kg-1). During the test, the child is encouraged to keep pedalling as fast as they can for a total of 30 s. Peak power (PP) output is calculated as the highest power output during the test, mean power (MP) is calculated as the average power over the 30 s. Since some participants may find it challenging to complete the full Wingate test, this study will also highlight the first 10 s as a ‘mini-Wingate’. This 10 s modified Wingate test has been shown to be feasible and reliable for preschoolers [24, 25]. MP over the first 10 s is calculated. All power calculations are measured in Watts (W) and will also be normalized to body mass (W/kg). Fatigue rates are measured after 10 s and 30 s using the minimum power output during the first 10 s and 30 s, respectively. Fatigue rate (%) is calculated as [(PP-min power)/PP) ×100].

For a secondary measure of lower body muscle strength, the child also completes a standing long jump. This measure is used in many field-based fitness assessments in young children [49, 50]. The child is asked to jump as far as possible from a standing position using a 2-foot takeoff and 2-foot landing. The child performs at least 3 attempts with the longest distance, measured to the nearest cm used to assess performance.

Flexibility is assessed using the Sit and Reach Test. The child sits on the floor with their feet held flat against the Sit and Reach Box (Figure Finder Flex-Tester, Novel Products Inc.) and reaches as far forward as they can while keeping both legs extended. Flexibility is measured to the nearest 0.5 cm and the better of two measurements is used. The Back-Saver Sit and Reach [51] protocol is also conducted. This is very similar to the traditional sit and reach test, except that only one leg at a time is assessed. A trial is excluded if the extended leg(s) bend(s) or the hands reach unevenly.

#### Physical activity

Accelerometry is an objective method of assessing free-moving physical activity, and is a well-established method for measuring activity in young children [52, 53]. The Actigraph GT3X+ activity monitor is a light (27 g) and small (3.8 cm × 3.7 cm × 1.8 cm) device. It is secured by a belt worn around the waist. The GT3X+ is capable of measuring accelerations from ~0.05 to 2 Gs and of capturing short epochs, allowing more accurate measurement of children’s physical activity [8, 54]. In line with existing recommendations, physical activity is monitored for 7 consecutive days [55] with removal of the device only when sleeping or prolonged exposure to water. Parents are asked to keep a diary, recording the time of day the accelerometer is placed on and taken off. Physical activity analysis will be conducted using ActiLife 6 software (ActiGraph, Pensacola, FL). Due to the short sporadic bursts of activity that are typical of young children, we will use 3 s epochs to accurately capture these short bouts of activity. The 2008 Evenson activity cut points for children will be used to determine levels of sedentary behaviour as well as time spent in light, moderate and vigorous intensity exercise. These established cut points have been validated in children aged 5–8 [56], and have been shown to have the highest accuracy in activity classification in children and adolescents aged 5–15 [57].

#### Questionnaires

Parents are asked to complete a questionnaire that asks questions about family demographics, measures of health and risk factors for obesity, and of participation in physical activity. In addition, a medical questionnaire is administered to confirm that the child has no contraindications to exercise. Parents also complete the Child Behaviour Checklist (CBCL) [58], which is used to assess behavioural (e.g. conduct problems), emotional problems (depression, anxiety) and autism-like behaviours [59]. The test-retest reliability of the CBCL scale scores range from 0.68 to 0.92 and reported classification accuracy ranges from 74 % to 84 % [60]. When appropriate (at age 6 years and older), parents will also complete the ADHD index of the Conners’ Parent Rating Scales, which covers diagnostic criteria in the DSM-IV [61]. Internal consistencies range from 0.85 to 0.94. Test-retest reliability ranges from 0.72 to 0.98 [61]. Given the young age of our cohort, we do not expect to see many cases of ADHD meeting DSM-IV diagnostic criteria at baseline. Thus, to obtain a reliable assessment of ADHD, we will administer the Conners’ twice over the course of the study (during years 2–4). Only children who meet criteria for ADHD based on the test [scores ≥6 (out of 9) on one or both subscales] at both times points will be identified as having ADHD. Each year the parents also complete the Developmental Coordination Disorder Questionnaire (DCD-Q), which is a measure used to identify motor coordination difficulties in children aged 5-15 [28].

#### Perceived self-efficacy

Perceived self-efficacy will be measured using the Perceived Efficacy and Goal Setting System (PEGS) [62]. PEGS uses colourful picture cards to illustrate essential tasks for participation in activities of daily living and school. These cards are presented to a child in pairs, with one picture demonstrating task competency and the other demonstrating less competence. The child then chooses which picture is more like him/her and then decides if he/she is ‘a lot’ or ‘a little’ like him/her. The scores for each item range from 1 (‘a lot like the less competent child’) to 4 (‘a lot like the more competent child’). The PEGS will be administered to children at all follow-up assessments.

#### Dietary intake

Parents complete a detailed food diary for 3 days (2 weekdays, 1 weekend day) recording the food and drinks consumed by their child for all meals and snacks noting brand names, quantities and preparation methods. The information provided will be analyzed to help interpret the relationship between body composition and dietary intake.

### Study protocol

#### Baseline testing – Phase 1

All eligible children are assessed in our laboratory to complete Phase 1 of baseline testing. At the baseline visit, parents complete all questionnaires and a semi-structured interview concerning the child’s ability to perform daily activities. The remainder of Phase 1 baseline testing includes assessments of motor skills (to determine presence of pDCD), cognitive ability (IQ), body composition, flexibility and standing long jump. Immediately following completion of the motor skill assessment, test results are scored and entered into a computer database. As mentioned previously, all children who score at or below the 16^th^ percentile on the M-ABC-2 as well as a random selection of children scoring above the 16^th^ percentile are invited into the longitudinal phase of the study and continue testing.

#### Baseline testing – Phase 2

For children selected to continue on to Phase 2 (i.e. longitudinal cohort), percent body fat, aerobic fitness, short-term muscle power output and foot structure are also assessed. At the end of Phase 2, an accelerometer to measure physical activity over the following 7 days is given to the child and any interested family members. An accelerometer logbook and 3-day food diary are also sent home with the family.

#### Testing years 2–4

All baseline assessments (excluding the IQ test) will be repeated once per year over the next 3 years. In addition, parents will complete the ADHD index (Conners) twice within the follow-up period and children’s self-efficacy to perform everyday tasks and activities will be assessed. In order to minimize attrition rates, we will provide parents with yearly reports that will highlight their child’s growth, physical activity, fitness and motor skills.

### Data analyses

To evaluate the stability of DCD over time, a straightforward descriptive analysis will be conducted. This will identify children whose scores change over time: those in the pDCD baseline group who, at a later assessment score above the 16^th^ percentile on the M-ABC-2 as well as those children in the control group whose measured performance declines to at or below the 16^th^ percentile, will be identified. At study end, children with 2 or more assessments differing from their baseline group assignment will be considered to have experienced substantial variability in motor proficiency. Depending on the numbers affected, they will either be used to form one or more additional groups in the analysis, or the analysis itself will be altered such that we retain baseline groups, but also model motor coordination as a time-varying characteristic.

To examine the association between DCD and fitness and adiposity, longitudinal growth models will be fitted with fixed effects for gender, baseline age, DCD status, ADHD symptoms (as these measures become available), time, and time by DCD. The outcomes will be total body fat, central body fat, aerobic fitness, and anaerobic fitness. First, these will be modeled as continuous, and then dichotomized at age-appropriate thresholds for overweight/obesity and inactivity, and fit additional models using the logistic link function. The time by group interaction will reveal the direction, magnitude, and significance of any divergence over time between the two groups. If we observe such a divergence, we will test whether it is mediated by physical activity by adding this measure to the above models. Finally, we will test the question of precedence using a distributed lag model in which each outcome is regressed on previous and contemporary measures of the predictors. This analysis will be repeated with a 3-group variable for DCD status that will differentiate among children who score at or below the 5^th^, those who score between the 6^th^ and the 16^th^, and those who score above the 16^th^ percentiles. This will allow us to assess whether the severity of motor impairment is associated with increased risk of poor fitness and overweight/obesity, and whether physical activity is a stronger mediator in the severe motor impairment group.

#### Testing secondary objectives

As noted above, we will adjust for covariates that may be associated with motor coordination, fitness and relative body weight. For example, we will estimate the models examining body fat and fitness with an index of ADHD obtained from the Conners’. Other covariates we will examine include perceived self-efficacy, diet, SES, maternal gestational diabetes and parental obesity. We will also examine developmentally important characteristics such as birth weight. Specifically, we will test for an association between DCD and low birth weight (1500–2500 g), and conduct a sensitivity analysis. Importantly, we will test for the potential moderating influence of sex on the association between DCD, inactivity, fitness and relative body weight. Specifically, we will repeat the analyses described in the preceding section, including a 3-way interaction (DCD by time by sex) interaction. This will allow us to ascertain if sex impacts these trajectories.

## Discussion

DCD is a highly prevalent chronic health condition, the impact of which has remained largely unrecognized. Given the significant number of children affected as well as how many of these same children show high rates of inactivity, poor fitness and obesity, it is essential that we better understand possible causal pathways between DCD and these aspects of physical health. To date, our studies have conclusively shown that many children identified with motor coordination problems in the general population are already unfit and overweight by the time they reach middle school. In order to fully understand these pathways, a longitudinal study beginning during a developmental period in which motor deficits are apparent but physical fitness has not yet deteriorated and obesity may not have yet developed, is required.

The CATCH study will be the first study to examine the longitudinal relationships among motor coordination, physical activity, fitness and adiposity from early to middle childhood.

It is essential to elucidate these relationships because the direction of these associations will help to determine the types of interventions children with DCD require. In some children, the presence of obesity and inactivity may lead to underdeveloped motor skills and poor fitness [63, 64]; for these children, interventions promoting physical activity and healthy diets may be appropriate. However, children with a diagnosis of DCD have fundamental motor impairments; thus, in these cases, a public health intervention targeting physical activity – the behaviour affected by the underlying disability – may be ineffective, and may even cause injury and/or be harmful to children’s self-esteem. For these children, a simultaneous effort must be made to adapt instructional methods and physical activity programs to accommodate their motor challenges [65]. It is therefore imperative that we understand the causal ordering in order to intervene in a manner that will address the actual problem.

The CATCH study will be the largest and most comprehensive study of children with DCD and health-related fitness conducted anywhere in the world and will be the first to examine the relationships among motor coordination, physical activity, physical fitness and adiposity in a prospective cohort of very young children. The knowledge generated from this study will not only answer fundamental questions related to pathways between DCD and health-related fitness, but will also generate knowledge useful for the design of targeted interventions.

## Abbreviations

DCD: Developmental Doordination Disorder
CATCH: Coordination and Activity Tracking in CHildren
BMI: Body mass index
pDCD: Probable DCD
DCD-Q: Developmental Coordination Disorder Questionnaire
M-ABC-2: Movement Assessment Battery for Children 2^nd^ Edition
KBIT-2: The Kaufman Brief Intelligence Test 2^nd^ Edition
WC: Waist circumference
BIA: Bioelectrical impedance analysis
FFM: Fat-free mass
FPI-6: Foot Posture Index Six-item version
HRR: Heart rate recovery
PP: Peak power
MP: Mean power

## References

[1] American Psychiatric Association (2000). Diagnostic and statistical manual of mental disorders, fourth edition, text revision. DSM IV-TR.

[2] Gibbs J, Appleton J, Appleton R (2007). Dyspraxia or developmental coordination disorder? Unraveling the enigma. Arch Dis Child.

[3] Missiuna C, Rivard L, Pollock N (2004). They’re bright but can’t write: developmental coordination disorder in school aged children. TEACHING Exceptional Children Plus.

[4] Henderson SE, Henderson L (2003). Toward an understanding of developmental coordination disorder: terminological and diagnostic issues. Neural Plast.

[5] Missiuna C, Moll S, King G, King S, Law M (2006). “Missed and misunderstood”: Children with coordination difficulties in the school system. Int J Spec Educ.

[6] Dewey D, Wilson B (2001). Developmental coordination disorder: What is it?. Phys Occup Ther Pediatr.

[7] Cairney J, Hay J, Veldhuizen S, Missiuna C, Faught B (2010). Developmental coordination disorder, sex, and activity deficit over time: a longitudinal analysis of participation trajectories in children with and without coordination difficulties. Devl Med and Child Neur.

[8] Green D, Lingam R, Mattocks C, Riddoch C, Ness A, Emond A (2011). The risk of reduced physical activity in children with probable developmental coordination disorder: a prospective longitudinal study. Res Dev Disabil.

[9] Bouffard M, Watkinson E, Thompson L, Causgrove Dunn J, Romanow S (1996). A test of the activity deficit hypothesis with children with movement difficulties. Adapt Phys Activ Q.

[10] Hands BD, Larkin D, Cermak S, Larkin D (2002). DCD and physical fitness. Developmental coordination disorder.

[11] Cairney J, Hay JA, Faught BE, Hawes R (2005). Developmental coordination disorder and overweight and obesity in children aged 9 to 14 y. Int J Obes (Lond).

[12] Faught BE, Hay JA, Cairney J, Flouris A (2005). Increased risk for coronary vascular disease in children with developmental coordination disorder. J Adolesc Health.

[13] Tsang WW, Guo X, Fong SM, Mak K-K, Pang MY (2012). Activity participation intensity is associated with skeletal development in pre-pubertal children with development coordination disorder. Res Dev Disabil.

[14] Cairney J, Hay JA, Faught BE, Flouris A, Klentrou P (2007). Developmental coordination disorder and cardiorespiratory fitness in children. Pediatr Exerc Sci.

[15] Schott N, Alof V, Hultsch D, Meermann D (2007). Physical fitness in children with developmental coordination disorder. Res Q Exerc Sport.

[16] Tsiotra GD, Nevill AM, Lane AM, Koutedakis Y (2009). Physical fitness and developmental coordination disorder in Greek children. Pediatr Exerc Sci.

[17] Zhu YC, Wu SK, Cairney J (2011). Obesity and motor coordination ability in Taiwanese children with and without developmental coordination disorder. Res Dev Disabil.

[18] Wagner MO, Kastner J, Petermann F, Jekauc D, Worth A, Bos K (2011). The impact of obesity on developmental coordination disorder in adolescence. Res Dev Disabil.

[19] Hands B (2008). Changes in motor skill and fitness measures among children with high and low motor competence: a five-year longitudinal study. J Sci Med Sport.

[20] Osika W, Montgomery S (2008). Physical control and coordination in childhood and adult obesity: longitudinal birth cohort study. BMJ.

[21] Cairney J, Hay J, Veldhuizen S, Faught BE (2011). Trajectories of cardiorespiratory fitness in children with and without developmental coordination disorder: a longitudinal analysis. Br J Sports Med.

[22] Cairney J, Hay J, Veldhuizen S, Missiuna C, Mahlberg N, Faught BE (2010). Trajectories of relative weight and waist circumference among children with and without developmental coordination disorder. CMAJ.

[23] Liu X, Spybrook J, Congdon R, Raudenbush S (2005). Optimal Design for Longitudinal and Multilevel Research, Version 1.55 HLM Software.

[24] Timmons B, Proudfoot N, MacDonald M, Bray S, Cairney J (2012). The health outcomes and physical activity in preschoolers (HOPP) study: rationale and design. BMC Public Health.

[25] Nguyen T, Obeid J, Timmons B (2011). Reliability of fitness measures in 3- to 5-year old children. Pediatr Exerc Sci.

[26] Parmar A, Kwan M, Rodriguez C, Missiuna C, Cairney J (2014). Psychometric properties of the DCD-Q-07 in children ages 4–6. Res Dev Disabil.

[27] Centre for Longitudinal Studies. National Child Development Study Questionnaire. www.cls.ioe.ac.uk/

[28] Wilson B, Crawford S, Green D, Roberts G, Aylott A, Kaplan B (2009). Psychometric properties of the revised developmental coordination disorder questionnaire. Phys Occup Ther Pediatr.

[29] Thompson W, Whittemore A, Evans A, Kelsey J (1996). Methods in Observational Epidemiology Second Edition.

[30] Blank R, Smits-Engelsman B, Polatajko H, Wilson P (2011). European Academy of Childhood Disability Recommendations (EACD): recommendations on the definition, diagnosis and intervention of developmental coordination disorder (long version). Dev Med Child Neurol.

[31] Henderson S, Sugden D, Barnett A (2007). Movement Assessment Battery for Children Examiner’s Manuel.

[32] Sugden DA (editor). Leeds Consensus Statement: Developmental Coordination Disorder as a Specific Learning Difficulty. Leeds:DCD-UK/Dyscovery Centre; 2006.

[33] Ellinoudi T, Evaggelinou C, Kourtessis T, Konstantinidou Z, Venetsanou F, Kambas A (2011). Reliability and validity of age band 1 of the movement assessment battery for children–second edition. Res Dev Disabil.

[34] Wuang Y, Su J, Su C (2012). Reliability and responsiveness of the movement assessment battery for children-Second Edition Test in children with developmental coordination disorder. Dev Med Child Neurol.

[35] Missiuna C, Gaines B, Soucie H (2006). Why every office needs a tennis ball: a new approach to assessing the clumsy child. CMAJ.

[36] Missiuna C, Pollock N, Egan M, DeLaat D, Gaines R, Soucie H (2008). Enabling occupation through facilitating the diagnosis of developmental coordination disorder. Can J Occup Ther.

[37] Kaufman A, Kaufman N (2004). Kaufman Brief Intelligence Test.

[38] Maffeis C, Pietrobelli A, Grezzani A, Provera S, Tato L (2001). Waist circumference and cardiovascular risk factors in prepubertal children. Obes Res.

[39] Goran M, Gower B, Treuth M, Nagy T (1998). Prediction of intra-abdominal and subcutaneous adipose tissue in healthy prepubertal children. In J Obes Relat Metab Disord.

[40] Taylor R, Williams S, Grant A, Ferguson E, Taylor B, Goulding A (2008). Waist circumference as a measure of trunk fat mass in children aged 3 to 5 years. Int J Pediatr Obes.

[41] Janz K, Witt J, Mahoney L (1995). The stability of children's physical activity as measured by accelerometry and self-report. Med Sci Sports Exerc.

[42] Watts K, Bell L, Byrne S, Jones T, Davis E (2008). Waist circumference predicts cardiovascular risk in young Australian children. J Paediatr Child Health.

[43] Wright C, Sherriff A, Ward S, McColl J, Reilly J, Ness A (2008). Development of bioelectrical impedance-derived indices of fat and fat-free mass for assessment of nutritional status in children. Eur J Clin Nutr.

[44] Goran M, Kaskoun M, Carpenter W, Poehlman E, Ravussin E, Fontvieille A (1993). Estimating body composition of young children by using bioelectrical resistance. J Appl Physiology.

[45] Villarroya M, Esquivel J, Tomas C, Beunafe A, Moreno L (2008). Foot structure in overweight and obese children. Int J Ped Obesity.

[46] Nieto L, Alegre L, Lain A, Vicen A, Casado M, Jodar A (2010). Does overweight affect the footprint and balance of school-aged children?. Apunts Med Esport.

[47] Redmond A, Crosbie J, Ouvrier R (2006). Development and validation of a novel rating system for scoring foot posture: the Foot Posture Index. Clin Biomech.

[48] van der Cammen-van M, IJsselstijn H, Takken T, Willemsen S, Tibboel D, Stam H (2010). Exercise testing of pre-school children using the Bruce treadmill protocol: new reference values. Eur J Appl Physiol.

[49] Rowland T, Rowland TW (1996). Body Composition. Developmental Exercise Physiology.

[50] Caspersen C, Powell K, Christenson G (1985). Physical activity, exercise, and physical fitness: definitions and distinctions for health-related research. Public Health Rep.

[51] Castro-Piñero J, Ortega F, Artero E, Girela-Rejón M, Mora J, Sjöström M (2010). Assessing muscular strength in youth: usefulness of standing long jump as a general index of muscular fitness. J Strength Cond Res.

[52] Meredith M, Welk G (2007). FITNESSGRAM/ACTIVITYGRAM: Test administration manual.

[53] Pate R, O’Neill J, Mitchell J (2010). Measurement of physical activity in preschool children. Med Sci Sports Exerc.

[54] Pate R, Almeida M, McIver K, Pfeiffer K, Dowda M (2006). Validation and calibration of an accelerometer in preschool children. Obesity (Silver Spring).

[55] Baquet G, Stratton G, Van PE, Berthoin S (2007). Improving physical activity assessment in prepubertal children with high-frequency accelerometry monitoring: a methodological issue. Prev Med.

[56] Trost S, Pate R, Freedson P, Sallis J, Taylor W (2000). Using objective physical activity measures with youth: how many days of monitoring are needed?. Med Sci Sports Exerc.

[57] Evenson K, Cattellier D, Gill K, Ondrak K, McMurray R (2008). Calibration of two objective measures of physical activity for children. J Sports Sci.

[58] Trost S, Loprinzi P, Moore R, Pfeiffer K (2011). Comparison of accelerometer cut points for predicting activity intensity in youth. Med Sci Sports Exerc.

[59] Achenbach T, Rescorla L (2000). Child behaviour checklist for ages 1 ½-5.

[60] Achenbach T, Rescorla L (2000). Manual for the ASEBA Preschool Forms & Profiles.

[61] Conners K (2008). Conners 3rd edition.

[62] Missiuna C, Pollack N, Law M (2004). Percieved Efficacy and Goal Setting System (PEGS).

[63] D Hondt E, Deforche B, De Bourdeaudhuij I, Lenoir M (2009). Relationship between motor skill and body mass index in 5- to 10-year old children. Adapt Phys Activ Q.

[64] Hume C, Okely A, Bagley S, Telford A, Booth M, Crawford D (2008). Does weight status influence associations between children's fundamental movement skills and physical activity?. Res Q Exerc Sport.

[65] Cairney J, Kwan M, King-Dowling S (2011). Obesity risk in children with Developmental coordination Disorder: What do we know? What can we do?. The Dyspraxia Fdn Prof J.

